# Natural-Origin Compounds as Future Precision Partners in Combination Cancer Therapy

**DOI:** 10.3390/medicina62071344

**Published:** 2026-07-12

**Authors:** Milica Pešić, Patricia Rijo, Natasa Z. Djordjevic

**Affiliations:** 1 Department of Neurobiology, Institute for Biological Research “Siniša Stanković”—National Institute of the Republic of Serbia, University of Belgrade, Despota Stefana 142, 11108 Belgrade, Serbia; 2 CBIOS—Research Center for Biosciences & Health Technologies, Universidade Lusófona, 1749-024 Lisbon, Portugal; patricia.rijo@ulusofona.pt; 3 Faculty of Pharmacy, Research Institute for Medicines (iMed.ULisboa), Universidade de Lisboa, 1649-003Lisbon, Portugal; 4 Department of Natural and Mathematical Sciences, State University of Novi Pazar, Vuka Karadzica 9, 36300 Novi Pazar, Serbia; natasadj@np.ac.rs

**Keywords:** cancer cells, multidrug resistance, ROS, natural products, collateral sensitivity, ferroptosis

## Abstract

Cancer multidrug resistance (MDR), particularly mediated by ATP-binding cassette (ABC) transporters, can link ABC transporters’ ATP-dependent efflux to Nrf2-driven antioxidant defence. This connection reduces the oxidative threshold in MDR cancer cells. Natural or nature-inspired compounds can target this vulnerability and induce collateral sensitivity (CS) by simultaneously modulating the redox balance and ABC transporters’ activity in MDR cancer cells. Moreover, natural-origin compounds can act on multiple targets by combining efflux inhibition, redox modulation, and immune evasion into a unique therapeutic strategy. However, many challenges should be addressed in their characterisation and preclinical validation to ensure their usefulness for clinical application. These include poor bioavailability, pharmacokinetic interactions, safe toxicity windows, and tumour heterogeneity. In perspective, adaptive trial designs employing biomarker-guided patient stratification can translate natural-origin compounds from preclinical promise to precision partners in clinical oncology.

## 1. Introduction

Cancer cells that are metabolically and chemically stressed balance at the edge of oxidative tolerance. To sustain their growth and rapid division, cancer cells generate reactive oxygen species (ROS) at levels far above those detected in normal tissues [1]. Increased ROS levels can drive cancer progression and, at the same time, be considered a vulnerability of cancer cells.

Multidrug-resistant (MDR) cancer cells may be more susceptible because ATP-binding cassette (ABC) transporters, usually expressed on the membrane of MDR cancer cells, consume large amounts of ATP to sustain drug efflux [2]. This demand for ATP elevates mitochondrial respiration and superoxide production, while Nrf2 antioxidant upregulation compensates by narrowing the cells’ oxidative safety margin [3]. This ATP-efflux-to-redox-imbalance link is currently a working model, largely derived from preclinical data, but it offers a rationale for a multi-level therapeutic strategy using natural-origin compounds. The simultaneous inhibition of ABC transporters and disruption of redox balance can induce collateral sensitivity (CS) in MDR cancer cells by exploiting a fitness cost created by resistance itself.

The natural chemodiversity represented by terpenoids and polyphenols from plants [4], marine products [5], microbial metabolites [6], and fungal compounds [7] provides a broad source with diverse natural scaffolds that can be harnessed to exploit MDR vulnerabilities and reframe therapeutic strategies in oncology. However, their exploitation is largely subjected to supply and standardisation constraints.

## 2. The Redox–MDR Relationship as a Targetable Vulnerability

ABC transporters are transmembrane proteins that couple ATP hydrolysis to the efflux of substrates. In cancer, three ABC transporters are predominantly responsible for resistance: ABCB1 (P-gp/MDR1), the best characterised, expels hydrophobic drugs such as taxanes, anthracyclines, and vinca alkaloids; ABCC1 (MRP1) exports anionic and glutathione conjugated metabolites including doxorubicin derivatives; and ABCG2 (BCRP) confers resistance to camptothecin derivatives, mitoxantrone, and topoisomerase II inhibitors via a half transporter homodimer mechanism [8,9].

Constant efflux activity, together with Nrf2-driven antioxidant upregulation and compensatory mitochondrial remodelling, forms an interdependent adaptive programme that sustains redox homeostasis in MDR cancer cells [10,11]. Disrupting any node of this programme can destabilise the balance that sustains MDR.

Besides the energetic coupling between ABC transporters’ activity and redox stress, Nrf2 also regulates ABC transporters’ expression at the transcriptional level [3]. This means that ABC transporter upregulation and antioxidant defence are under shared transcriptional control and that although oxidative stress governs MDR cancer cells’ vulnerability, targeting Nrf2 strengthens the rationale for simultaneously suppressing ABC transporters’ efflux capacity and the oxidative buffering capacity of MDR cancer cells.

More than half of approved anticancer agents derive from natural sources [12], and many act precisely on redox homeostasis. The historical route by which natural compounds have been widely used in oncology was largely serendipitous. The modern discovery approach is guided by mechanism-related and cancer-specific vulnerability profiling. Therefore, nature-inspired compounds can advance precision oncology when selected for their ability to destabilise redox balance in resistant cells and when combined with other anticancer drugs. Compounds that suppress Nrf2, deplete glutathione (GSH), or trigger mitochondrial ROS disrupt this circuit more effectively than the three generations of efflux inhibitors that do not act on redox balance [13].

## 3. Diterpenoids and Mitochondrial Targeting

Several mechanistic paths illustrate this principle. Abietane diterpenoids from *Plectranthus* spp. retain cytotoxic potency in P-gp-overexpressing cancer cells through an ROS-dependent, mitochondria-mediated apoptotic mechanism [14]. Coleon U, another diterpenoid from *Plectranthus* spp., inhibits mitochondrial electron transport and reduces ATP for ABCB1 efflux while increasing ROS, thereby creating a dual vulnerability in MDR cancer cells that is hard to escape [15].

## 4. Semisynthetic Hybrids and Collateral Sensitivity

**Figure 1:**
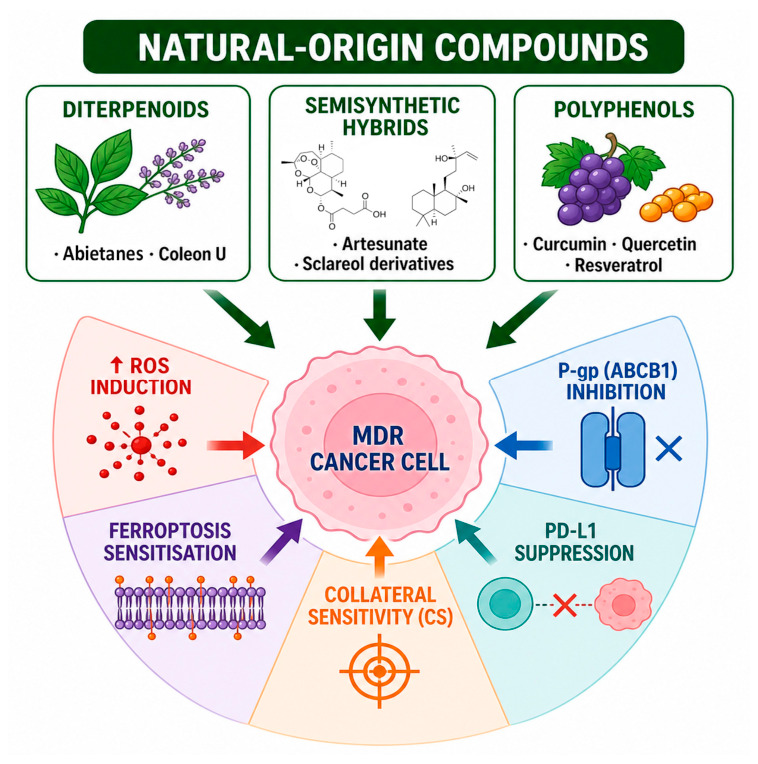
Natural-origin compounds and their mechanisms of action in MDR in cancer cells. Different nature-derived or inspired classes of compounds, exemplified by diterpenoids (Abietanes, such as Coleon U), semisynthetic hybrids (Artesunate, Sclareol derivatives), and polyphenols (Curcumin, Quercetin, Resveratrol), can exploit some of the vulnerabilities of MDR cancer cells. (1) Increased ROS generation exhausts antioxidant defences, promoting oxidative stress-mediated cell death. (2) The disruption of lipid peroxide homeostasis and the GSH/GPX4 axis restores sensitivity to ferroptosis. (3) Collateral sensitivity-The MDR phenotype that confers resistance to anticancer drugs, in some cases, leads to hypersensitivity to natural-origin compounds. (4) The suppression of PD-L1 enhances antitumour immune responses. (5) The inhibition of P-gp activity restores intracellular drug accumulation and reverses the MDR phenotype. These distinct mechanisms make natural-origin compounds promising for combination strategies in MDR tumours, in which ABC inhibitors have failed to achieve a significant effect on patient survival.

Semisynthetic hybrids represent a sophisticated approach to MDR circumvention by inducing CS in MDR cancer cells. CS is the phenomenon in which the acquisition of MDR confers delicate vulnerability to a mechanistically distinct class of agents, thereby representing an exploitable fitness cost inherent to resistance [16]. The molecular basis of CS in MDR cancer cells was established through the demonstration that P-gp overexpression weakens antioxidative capacity: MDR cancer cells exhibit lower basal GSH levels, downregulated manganese superoxide dismutase (MnSOD) and glutathione-S-transferase π (GST π), and elevated vulnerability to exogenous H_2_O_2_ relative to their sensitive counterpart cells [17]. Protoflavones were identified as a natural scaffold class for selectively exploiting this imbalance by differentially modulating ROS/RNS in MDR versus non-MDR cancer cells, downregulating HIF-1α, and producing an inverse sensitivity-versus-MDR selectivity pattern across multiple P-gp-overexpressing cell lines [17]. This redox–CS framework was also demonstrated across structurally diverse semisynthetic scaffolds. Artesunate, with the potential to inhibit P-gp function [18], induces ROS-mediated apoptosis via the intrinsic mitochondrial pathway, where NAC blockable H_2_O_2_ accumulation overcomes doxorubicin resistance in T-cell leukaemia through a mechanistically distinct killing route from doxorubicin [19]. Artesunate–pyrimidine hybrids exploit differential redox balance between sensitive and resistant non-small cell lung carcinoma cells to achieve CS indexes up to 5, additionally inhibiting P-gp activity more potently than the benchmark inhibitor tariquidar [20]. Sclareol–doxorubicin hybrids self-assemble into nanoparticles, demonstrate activity in P-gp-expressing MDR glioblastoma cells, and induce mitochondrial membrane depolarisation and oxidative stress [21]. Sclareol–adamantane derivatives similarly self-assemble into uniform, positively charged nanoparticles that reverse doxorubicin resistance in MDR non-small cell lung carcinoma cells [22], while the most potent hybrids were shown to exploit ROS vulnerability to overcome MDR in glioblastoma cells [23]. Collectively, these studies show that the differential antioxidative capacity between sensitive and resistant cells is the key determinant of CS induction by natural and semisynthetic scaffolds, while the simultaneous targeting of P-gp provides an MDR-overcoming mechanism that resistant cancer cells cannot readily adapt to (Figure 1, Table 1), especially when CS agents are combined with partner anticancer drugs.

## 5. Ferroptosis Induction and Nrf2 Suppression

Resistance to approved natural-origin drugs generates metabolic and redox vulnerabilities that other natural compounds can exploit to sensitise MDR cancer cells. Paclitaxel resistance involves P-gp efflux, β-tubulin mutations, and cytoskeletal reorganisation, all the adaptations that are collectively ATP-costly and ROS-generating, priming resistant cells for oxidative challenge [24,25]. Doxorubicin resistance further involves MDR Protein 1 (MRP1) upregulation and the dysregulation of ceramide signalling [26,27], leading to a simultaneous elevation in ROS and mitochondrial stress [28], a state that increases vulnerability to pro-oxidant co-treatment. Camptothecin and its derivatives are prone to ABCG2-mediated efflux as a primary mechanism of resistance [29]. Flavonoids can efficiently address resistance developed to camptothecin through a dual mechanism: the direct competitive inhibition of ABCG2-mediated transport [30] and pro-oxidant redox modulation that selectively amplifies ROS in tumour cells and disrupts mitochondrial membrane potential [31]. This dual action positions flavonoids as sensitisers that simultaneously restrict drug export and exploit the bioenergetic vulnerabilities that resistance itself creates.

A major development is ferroptosis, an iron-dependent form of cell death driven by lipid peroxidation when GPX4 fails [32]. Because it bypasses apoptosis signalling, ferroptosis circumvents classical MDR. Natural compounds such as artesunate, sulforaphane, quercetin, gliotoxin, and timosaponin AIII act as ferroptosis inducers, exploiting the fact that MDR cancer cells are already near the ferroptotic threshold [33]. Targeting Nrf2 undermines both ferroptosis resistance and P-gp efflux, connecting two resistance programmes through a single molecular axis. For example, curcumin, as a single natural compound, can overcome multiple mechanisms of resistance (Figure 1, Table 1). It suppresses Nrf2-driven antioxidant transcription [13], downregulates P-gp expression and function [34], and shifts the Bcl-2/Bax ratio towards apoptosis [13], thus restoring sensitivity to 5-fluorouracil in MDR colorectal cancer [35] and to paclitaxel in MDR breast cancer [36].

## 6. Synergy with Targeted Therapies and Immunotherapy

Natural compounds also synergise with targeted therapies and immunotherapies. Resistance to EGFR TKIs, such as osimertinib, involves kinase bypass pathways that overlap with Nrf2 signalling [37,38]. Polyphenols downregulate PD-L1 via PI3K-AKT, JAK-STAT, and NF-κB [39], while resveratrol–curcumin–quercetin combinations remodel the tumour microenvironment, reducing regulatory T-cell infiltration [40]. Compounds that simultaneously downregulate P-gp, suppress PD-L1, and induce ferroptosis offer multi-axis synergy (Figure 1, Table 1).

Harnessing the full potential of natural-origin compounds in medical treatments demands a well-structured approach. Given the rich biodiversity of our planet and the unexplored chemodiversity, ROS-active compounds can be isolated from diverse sources, including marine organisms, plants, and microorganisms. Following isolation, these compounds can be further enhanced using semisynthetic methods, ensuring they function optimally in cancer cells. Next, mechanism-focused phenotyping should be applied to explore their actions and validate them in patient-derived organoids (PDOs), specifically targeting biomarkers such as Nrf2, GSH, GPX4, and P-gp.

## 7. Translational Challenges

Important challenges must be addressed to secure the clinical translation of natural-origin compounds. The variability in content within the same source and across broad geographical distributions, seasonal changes, and exposures to different environmental conditions requires the standardisation of isolation methods.

Bioavailability represents another significant barrier. Upon oral administration, curcumin has low systemic availability [41], and nano-formulations such as liposomes, micelles, and polymeric nanoparticles have been developed to enhance curcumin absorption and tissue penetration.

Pharmacokinetic interactions should also be carefully considered. Redox modulators, when used in combination, can alter the metabolism of partnering anticancer drugs. For instance, quercetin inhibits CYP3A4 [42] and therefore can change drug disposition in combination strategies. Safety profiling is also important. Pro-oxidant compounds risk harming normal tissues that possess high ROS levels, such as the intestinal epithelium and cardiomyocytes. The cardiotoxicity of doxorubicin is well-documented [43]. This can set a narrow therapeutic window for any co-administered pro-oxidant agent.

In addition, tumour heterogeneity complicates predictions of efficacy. The MDR phenotype is not uniform. Distinct populations within a single tumour may exhibit divergent Nrf2 and P-gp expression profiles, leading to inconsistent responses. Functional diagnostics as a response prediction tool and biomarker-guided patient selection, focused on Nrf2, GSH, GPX4, and P-gp status, as a patient stratification tool could be essential for identifying the patients most likely to benefit from redox-exploiting combination strategies with natural-origin compounds.

The mechanistic evidence underpinning redox-exploiting strategies is limited because it is derived almost entirely from in vitro results (Table 1). Although the results are promising, they cannot capture pharmacokinetics, systemic immune contributions, or the 3D tumour microenvironment, which are necessary to determine preclinical efficacy. Closing this gap requires 3D PDO and organoid-on-chip co-cultures with stromal and immune components to confirm that ROS/Nrf2/P-gp-dependent CS occurs under physiologically relevant conditions. The FDA Modernization Act 2.0 as standalone preclinical evidence now accepts these platforms [44]. In parallel, the use of physiologically based pharmacokinetic modelling can identify the risks of bioavailability and CYP-mediated interactions before starting animal studies, thereby focusing only on candidates with a favourable predicted exposure profile. Then, lead compounds should be tested in patient-derived xenograft models, which preserve the genetic and stromal heterogeneity of the original tumour, allowing CS- and ferroptosis-inducing efficacy to be assessed in vivo. This sequential pipeline is necessary to address the in vitro-only evidence currently summarised in Table 1.

## 8. Future Research Priorities

The standardised seasonal and geographical sourcing of materials from nature to minimise variability, combined with HPLC purity assessment, is essential to ensure the quality of isolated natural structures and their comparability across studies [45].

Physiologically based pharmacokinetic modelling should be employed to predict the behaviour of natural-origin compounds and their interactions with partner anticancer drugs in combination therapies. This approach simulates absorption, distribution, metabolism, and elimination across virtual populations and is gaining acceptance by regulatory agencies for oncology drug applications [46]. Applying it at the early stages of research will help reduce dependence on empirical in vivo testing for clinical trial entry.

PDOs are increasingly used as a platform for functional diagnostics. The identification of responsive cell populations within PDOs can be achieved by combining functional diagnostics with the assessment of Nrf2 and P-gp expression profiles to enhance the translational value of in vitro results using natural-origin compounds [47].

Using natural-origin compounds in clinical settings requires strict manufacturing standards and robust safety assessments to meet regulatory requirements and support mechanistic studies. Upon meeting these standards, adaptive trial designs that link compound activity to tumour biology will also facilitate the transition from standard combination chemotherapy to biomarker-driven precision therapy for patients with MDR cancer.

## 9. Conclusions

Natural-origin compounds could be considered precision tools for exploiting the oxidative vulnerabilities of MDR cancer cells in the future. In this way, natural-origin compounds will depart from being only traditional medicine. When lead compounds are carefully selected for their ROS and ABC transporter-modifying activity, optimised through semisynthetic derivatisation, and validated in PDO models, natural-origin compounds can challenge resistance mechanisms that many approved anticancer drugs fail to overcome. Embedding them within adaptive, biomarker-guided trials will complete their transition to precision partners in combination anticancer therapy.

## Figures and Tables

**Table 1:** Natural-origin compound examples, their mechanisms, current level of evidence, and limitations.

Compound Class	Examples	Targets	Mechanism in MDR Cells	Preclinical Evidence	Limitations
Abietane diterpenoids	Coleon U, Parvifloron D	P-gp, mitochondria	ATP depletion → P-gp inhibition; ROS ↑ → apoptosis	In vitro	Single cell line
Artesunate hybrids	Artesunate–pyrimidine	P-gp, ROS	CS induction; P-gp inhibition > tariquidar	In vitro	No in vivo confirmation
Sclareol hybrids	Sclareol–doxorubicin, Sclareol–adamantane	P-gp, mitochondria	Nanoparticle self-assembly; mitochondrial depolarisation; ROS ↑	In vitro	CMC complexity
Polyphenols	Curcumin, Resveratrol	Nrf2, P-gp, PD-L1	Nrf2 suppression; ferroptosis restoration; immune modulation	In vitro In vivo	Poor oral bioavailability
Flavonoids	Quercetin	ABCG2, ROS	Competitive ABCG2 inhibition + pro-oxidant modulation	In vitro	CYP3A4 interaction risk

## Data Availability

No new data were created or analyzed in this study.

## Abbreviations

ABC	ATP-binding cassette
AKT	Ak strain transforming or protein kinase B (PKB)
ATP	Adenosine triphosphate
Bax	Bcl-2–associated X protein
Bcl-2	B-cell lymphoma 2
BCRP	Breast cancer resistance protein
CMC	Chemistry, manufacturing, and controls
CS	Collateral sensitivity
CYP	Cytochrome P450 enzyme
EGFR	Epidermal growth factor receptor
GPX4	Glutathione peroxidase 4
GST	Glutathione S-transferase
H_2_O_2_	Hydrogen peroxide
HIF-1α	Hypoxia-inducible factor 1 subunit alpha
HPLC	High‑performance liquid chromatography
JAK	Janus kinase
MDR	Multidrug resistance
MnSOD	Manganese superoxide dismutase
NAC	N-acetylcysteine
NF-κB	Nuclear factor kappa-light-chain-enhancer of activated B cells
Nrf2	Nuclear factor erythroid 2-related factor 2
PD-L1	Programmed death-ligand 1
PDO	Patient-derived organoid
P-gp	P-glycoprotein
PI3K	Phosphoinositide 3-kinase
RNS	Reactive nitrogen species
ROS	Reactive oxygen species
STAT	Signal transducer and activator of transcription
TKI	Tyrosine kinase inhibitor
